# First Nearly Complete Genome Sequence of *Feline immunodeficiency virus* from Brazil

**DOI:** 10.1128/genomeA.00947-17

**Published:** 2017-09-28

**Authors:** Sueli A. Taniwaki, João P. Araújo, Paulo E. Brandão

**Affiliations:** a Department of Preventive Veterinary Medicine and Animal Health, School of Veterinary Medicine, University of São Paulo, São Paulo, São Paulo, Brazil; b Department of Microbiology and Immunology, Institute of Bioscience, São Paulo State University (UNESP), Botucatu, São Paulo, Brazil; c Institute for Biotechnology (IBTEC), São Paulo State University (UNESP), Botucatu, São Paulo, Brazil

## Abstract

*Feline immunodeficiency virus* (FIV) has worldwide distribution; nevertheless, only a few FIV genomes from domestic cats are available. This is the first report of a nearly complete genome of FIV from a Brazilian cat (8,967 nucleotides [nt]), including the entire coding region and the 3′ untranslated region.

## GENOME ANNOUNCEMENT

*Feline immunodeficiency virus* (FIV) infection is one of the most impactful infectious diseases of domestic cats, causing immunological dysfunction associated with opportunistic infections in a fashion similar to AIDS in humans infected with HIV. FIV is a member of the *Retroviridae* family, *Orthoretrovirinae* subfamily, *Lentivirus* genus, and is classified into five well-characterized subtypes (A to E) based on *gag* and *env* genes (1–3). Subtype B FIV appears to be more adapted to the host, causing a longer asymptomatic stage, with mild or no clinical signs. This lower virulence is related to differences in genome sequences probably acquired during coevolution (4). Partial sequences of the *gag*, *pol*, and *env* genes demonstrated that only subtype B is detected in Brazilian cats, but no complete genome is available (5).

The proviral DNA was extracted from whole blood of a naturally infected cat and amplified with AccuTaq LA DNA polymerase (Sigma-Aldrich). The ca. 9.0-kb amplicon was purified and submitted to library construction with a Nextera XT index kit and Nextera XT DNA kit (Illumina). The library was sequenced using MiSeq reagent kit version 3 (150 cycles) and the MiSeq system platform (Illumina) and generated 422,482 reads. Reads were trimmed and assembled with CLC Genomics Workbench version 9.5.3 (Qiagen). *De novo* assembly of 421,117 reads after trimming (quality limit, 0.05) generated a long contig with 9,005 nucleotides (nt) using 405,313 reads and with 5,413-fold average coverage. The primer sequences were removed, and phylogenetic analyses were conducted with MEGA 6 (6) and BioEdit Sequence Alignment Editor (7) softwares.

The nearly complete FIV genome sequence of 8,967 nt includes the entire coding region of structural proteins Gag (nt 253 to 1599), Pol (nt 1491 to 4862), and Env (nt 5885), accessory proteins Vif (nt 4855 to 5610) and open reading frame A (ORF A) (nt 5611 to 5847), and a 3′ untranslated (U3) region (nt 8730 to 8944). A comparison with the only seven complete FIV genome sequences from domestic cats available on GenBank confirmed the presence of subtype B in Brazil. Compared with the subtype B TM2 strain (GenBank accession number M59418), the full genome showed 95.4% nucleotide identity and 97.1, 96.2, 95.2, 96.0, and 91.0% amino acid identities, respectively, to proteins Gag, Pol, Env, Vif, and ORF A.

This is the first description of a nearly complete genome sequence of a Brazilian FIV strain and adds significant data to the hitherto few available complete FIV genomes, despite the lack of the 5′ untranslated (U5) region. The 5′ and 3′ long terminal repeats (LTRs) of retrovirus have the same U3RU5 (R indicates a repeat region) sequence, and the U3 region contains sequences that interact with cellular enhancer/promoter binding proteins (8). Although only partial genomic sequences are widely used, mainly the V3-V5 hypervariable regions of *env*, a recent study showed higher diversity in the *env* leader sequence (9). The availability of U3 and the entire coding region of FIV allows for an improvement of the knowledge on the epidemiology, diversity, and evolution of this virus.

### Accession number(s).

The nearly complete genome sequence of *Feline immunodeficiency virus*/cat/Brazil/Pequeno_2013 has been deposited in GenBank under the accession number MF370550.
